# Gaze-Evoked Vomiting After Minor Orbital Trauma: White-Eyed Blowout Fracture

**DOI:** 10.5811/cpcem.65614

**Published:** 2026-09-08

**Authors:** Naoki Yamada, Yukinori Kato

**Affiliations:** * University of Fukui Hospital, Department of Emergency Medicine and General Medicine, Fukui, Japan; † University of Fukui Hospital, Department of Otorhinolaryngology–Head and Neck Surgery, Fukui, Japan

**Keywords:** white-eyed blowout fracture, orbital trauma, trapdoor fracture, oculocardiac reflex, diplopia

## Abstract

**Case Presentation:**

A 16-year-old girl presented to the emergency department after being struck in the right eye by another player’s elbow during a basketball game. She had only minimal periorbital swelling but developed persistent nausea, repeated vomiting, and diplopia with upward gaze. Visual acuity, visual fields, and pupillary light reflexes were normal. Extraocular movement testing showed mild limitation of upward gaze of the right eye, and upward gaze reproducibly provoked diplopia, marked nausea, and vomiting. Computed tomography (CT) showed no intracranial hemorrhage, and orbital CT was initially interpreted as showing no obvious orbital floor fracture or extraocular muscle entrapment. Given the discrepancy between the mild external appearance and the severe ocular symptoms, white-eyed blowout fracture with trapdoor-type entrapment and an oculocardiac reflex was suspected. She underwent emergent surgery, after which her vomiting, diplopia, and ocular motility disturbance improved immediately.

**Discussion:**

White-eyed blowout fracture is a trapdoor-type orbital floor injury seen mainly in children and adolescents and may present with minimal external signs and subtle or negative-appearing CT findings.^1^–^3^ Gaze-evoked nausea or vomiting, diplopia, and relative bradycardia are important bedside clues to extraocular muscle entrapment with an oculocardiac reflex.^1^–^3^ This case highlights that white-eyed blowout fracture may be detected by careful bedside examination even when CT does not clearly show an orbital floor fracture. Emergency physicians should recognize this entity as a clinically important, easily missed diagnosis in pediatric orbital trauma.

## CASE PRESENTATION

A 16-year-old previously healthy female was brought to the emergency department after being struck in the right periorbital region by an opponent’s elbow during a basketball game. She was alert and hemodynamically stable: blood pressure, 126/73 mm Hg; heart rate, 56 beats per minute; respiratory rate, 18 breaths per minute; oxygen saturation, 99% on room air; and temperature, 36.8 ºC. Examination showed only minimal swelling around the right eye without marked ecchymosis. Visual acuity, visual fields, and pupils were normal. Extraocular movement testing showed mild limitation of upward gaze of the right eye. Upward gaze reproducibly provoked diplopia, marked nausea, and repeated vomiting. Head computed tomography (CT) showed no traumatic intracranial findings. Orbital CT was initially interpreted as showing no obvious orbital floor fracture or extraocular muscle entrapment (Image 1).

Despite the mild external appearance and initially subtle imaging findings, the combination of gaze-evoked vomiting, diplopia, mild upward gaze limitation, and relative bradycardia raised concern for a white-eyed blowout fracture with trapdoor-type entrapment and an oculocardiac reflex. Otolaryngology was urgently consulted, and the patient underwent emergent surgery. Intraoperatively, a right orbital floor fracture with suspected orbital soft-tissue entrapment was identified and released (Image 2). Her vomiting, diplopia, and ocular motility disturbance improved promptly after surgery.

## DISCUSSION

White-eyed blowout fracture is a trapdoor-type orbital floor injury seen in children and adolescents.^1^–^3^ Pediatric facial fractures account for less than 15% of all facial fractures, and orbital blowout fractures constitute approximately 20% of pediatric facial fractures.^3^ In trapdoor fractures, the elastic orbital floor may transiently open and recoil, entrapping the inferior rectus muscle or adjacent orbital soft tissue while leaving only minimal external signs of trauma.^1^–^3^ Consequently, clinicians may miss the diagnosis when swelling or ecchymosis is minimal.

This case emphasizes that CT may underestimate or fail to clearly demonstrate pediatric trapdoor-type injury.^2^ Although CT is the first-line imaging modality for orbital trauma, soft-tissue entrapment may not be apparent on initial interpretation. Parbhu et al reported a radiologic-intraoperative concordance rate of 50% in children with orbital floor fractures, compared with 87% in adults.^2^ Therefore, extraocular muscle entrapment should be considered a clinical diagnosis rather than a purely radiographic one.

In young patients with orbital trauma, the mismatch between mild external findings and severe symptoms is an important clue. Diplopia, restricted upward gaze, nausea or vomiting triggered by eye movement, and relative bradycardia should raise concern for extraocular muscle entrapment with an oculocardiac reflex.^1^–^4^ Early recognition is important because delayed release of entrapped tissue may lead to ischemia, fibrosis, persistent diplopia, and ocular motility dysfunction.^1^,^3^ Missed diagnosis and pediatric trapdoor fractures with subtle CT findings have been reported previously.^4^,^5^ This case adds to the emergency medicine literature by highlighting gaze-evoked vomiting as a bedside clue to white-eyed blowout fracture when CT findings are subtle or initially interpreted as negative.

*CPC-EM Capsule*

What do we already know about this clinical entity?

*White-eyed blowout fracture can present with minimal external signs but cause extraocular muscle entrapment and oculocardiac symptoms.*

What is the major impact of the image(s)?

*The images demonstrate subtle computed tomography (CT) findings and operative confirmation of trapdoor entrapment despite minimal external trauma signs.*

How might this improve emergency medicine practice?

*Emergency physicians should suspect trapdoor fracture when diplopia, vomiting, or bradycardia follows minor orbital trauma, even with subtle CT findings.*

## Figures and Tables

**Image 1:**
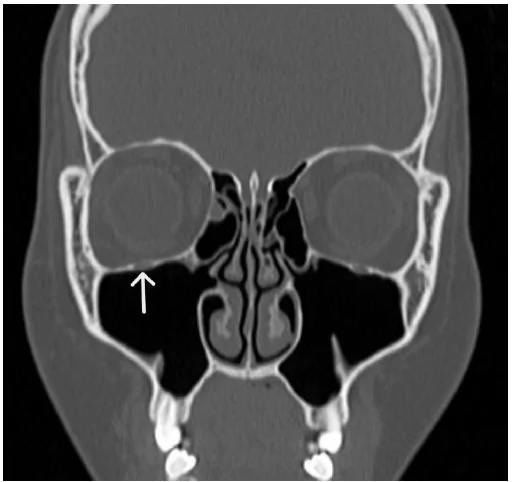
Coronal orbital computed tomography at presentation. The arrow indicates the apparently intact right orbital floor, with no visible evidence of fracture or extraocular muscle entrapment.

**Image 2:**
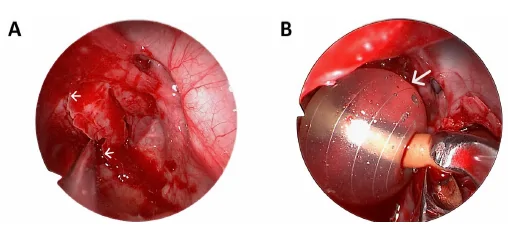
Endoscopic transnasal surgical findings. A: Intraoperative endoscopic view demonstrating the orbital floor fracture line, indicated by white arrows. A forceps is advanced toward the fracture cleft. B: After release and reduction of the prolapsed orbital tissues, a balloon catheter, indicated by a white arrow, is placed to support the repaired orbital floor.
